# Availability of the National Diabetes Prevention Program in United States Counties, March 2017

**DOI:** 10.5888/pcd15.180063

**Published:** 2018-09-06

**Authors:** Bina Jayapaul-Philip, Shifan Dai, Karen Kirtland, Alyson Haslam, Kunthea Nhim

**Affiliations:** 1 Division of Diabetes Translation, Centers for Disease Control and Prevention, Atlanta, Georgia; 2 CyberData Technologies, Inc., Atlanta, Georgia; 3 Northrop Grumman, Atlanta, Georgia; 4 Oregon Health & Science University, Portland, Oregon

**Figure:**
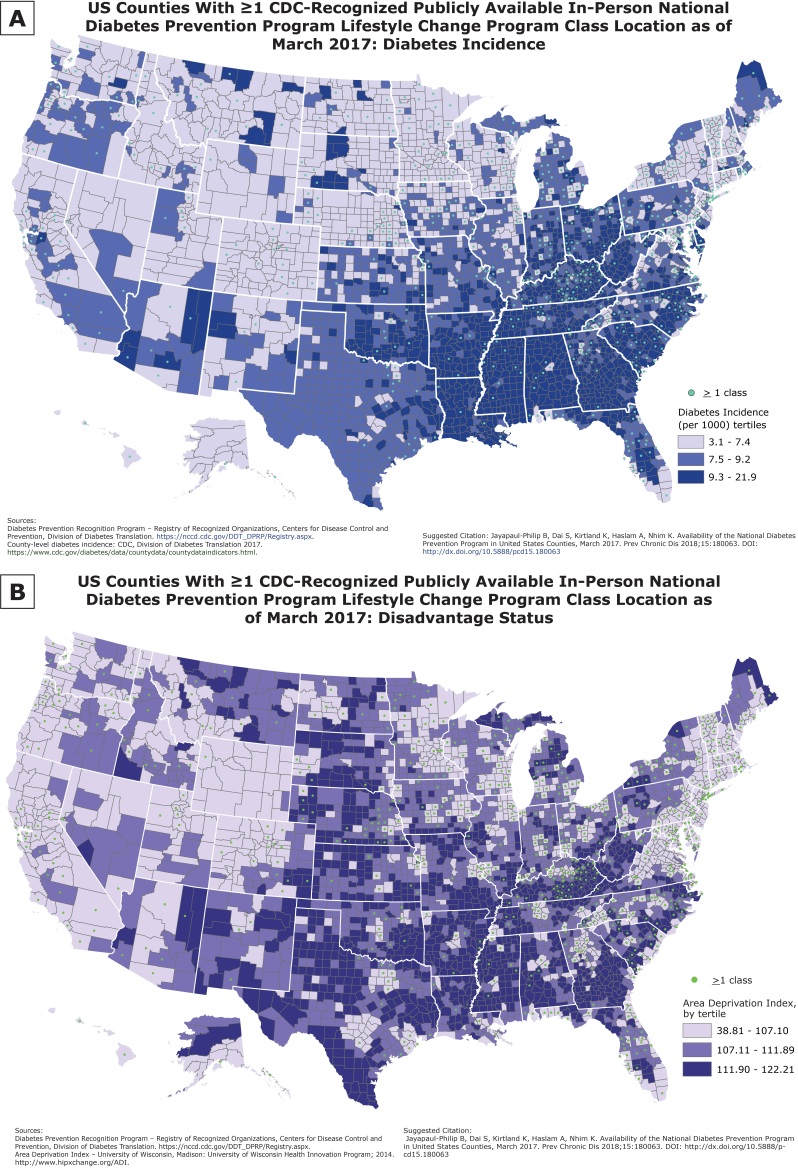
These maps show publicly available in-person lifestyle change program (LCP) classes, as of March 1, 2017, by diabetes incidence (Map A) and socioeconomic status (Map B) at the county level. Because higher diabetes incidence is correlated with lower socioeconomic status, this information may be useful in targeting type 2 diabetes prevention efforts. Organizations wanting to expand the availability of the LCP may use these maps to determine counties most in need of new programs.

## Background

In the United States, 84.1 million adults are estimated to have prediabetes, a serious health condition in which blood sugar levels are higher than normal but not high enough for a diagnosis of diabetes (1). Prediabetes increases the risk for type 2 diabetes, heart disease, and stroke (1). Through the Centers for Disease Control and Prevention (CDC)-led National Diabetes Prevention Program (National DPP), people with prediabetes can learn to make practical, real-life changes that can reduce their risk for developing type 2 diabetes by as much as 58% (71% for people aged ≥60 years) (1). CDC is working to expand the lifestyle change program (LCP) across the country, via the National DPP (2). Given the large number of people affected by prediabetes, CDC has several efforts to increase the availability of the National DPP LCP including Cooperative Agreements such as “Scaling the National Diabetes Prevention Program in Underserved Areas” (DP17-1705). We assessed the presence of publicly available in-person LCP classes, as of March 1, 2017, by diabetes incidence and socioeconomic status at the county level, because higher diabetes incidence and lower socioeconomic status are correlated (3) and may be useful in targeting type 2 diabetes prevention efforts. Organizations wanting to expand the availability of the LCP may use these maps to determine counties most in need of new programs.

## Data Sources and Map Logistics

We chose the mapping strategy to visualize areas that need additional programs, especially in light of high diabetes incidence and socioeconomic disadvantage. Visualization by using maps enables deeper insights into the data, such as patterns and relationships that can inform program planning decisions.

### Geocoding of LCP locations and map development

We used a list of addresses of publicly available in-person LCP classes (77%) or CDC-recognized organizations if the address of the class location was not available (23%) from the Diabetes Prevention Recognition Program (DPRP) as of March 1, 2017. Organizations had achieved either full or pending CDC recognition status. We geocoded addresses to counties by using R (version 3.3.2). We used the US Census geocoder to provide information for locations that did not geocode originally. We classified US counties with at least 1 census tract that had one or more LCP class locations as counties with LCP classes, represented by a centroid. We mapped a total of 1,558 LCP class locations. We used ArcGIS 10.0 (Esri) to join data for diabetes incidence, Area Deprivation Index, and location of LCP classes with a geographic boundary shape file for the 3,142 counties and county equivalents in the United States by using the 5-digit Federal Information Processing Standards codes for counties.

### Diabetes incidence

The most recent data available for county-level age-adjusted incidence rates for diagnosed diabetes as of November 2017 were obtained from CDC (4). We classified counties on the basis of low (3.1 – 7.4), medium (7.5 – 9.2), and high (9.3 – 21.9) diabetes incidence according to tertiles of incidence rates, to have roughly equal groupings of counties and to easily discern the distribution pattern.

### Socioeconomic status of counties

We used the Area Deprivation Index (ADI) both for its comprehensiveness as a composite score that captures various indicators of socioeconomic status and because of its use in recent analyses that consider contextual factors predictive of health outcomes. ADI represents a geographic area–based measure of the socioeconomic disadvantage experienced by a community and includes variables such as level of education, housing value, family income, and unemployment status (5). Higher index values represent higher levels of disadvantage. High disadvantage is associated with a higher risk for poor health and poor healthcare outcomes (6). We used an updated and validated census block group ADI, based on 2009–2013 American Community Survey 5-year estimates, from the University of Wisconsin (see Acknowledgments). We calculated the weighted average ADI, by census block group population, for each US county and divided the counties into tertiles based on their ADI scores.

## Main Findings

LCP classes were located in 711 (23%) US counties as of March 1, 2017. The maps show clustering of LCPs in the lighter shaded areas, which represent counties with low diabetes incidence and high socioeconomic status. This pattern and the percentage distributions are shown in the Table. More counties in the lowest tertile of diabetes incidence (26.8%) had an in-person LCP class location, whereas fewer counties in the middle and high diabetes incidence tertile (23.7% and 17.4%, respectively) had a class location. More counties (39%) in the lowest ADI tertile had an LCP class location than did counties in the middle and highest ADI tertiles (19.3% and 9.7%, respectively). Counties with the middle and highest ADI tertiles have the greatest economic need.

**Table:** Percent of US Counties With at Least 1 CDC-Recognized Publicly Available In-Person Lifestyle Change Program Class Location by County-Level Diabetes Incidence (2013) and Area Deprivation Index, March 1, 2017

Category	Low Tertile (%)	Medium Tertile (%)	High Tertile (%)
**Diabetes Incidence**	26.8	23.7	17.4
**Area Deprivation Index**	39.0	19.3	9.7

Several caveats relate to the information provided here. This analysis is based on DPRP data as of March 2017; there may be many more class locations now than are depicted here. CDC-recognized organizations were not required to provide class location information; additional class locations may have been available at the time. Some counties are physically larger than others, and people may attend classes in neighboring counties. There are also online LCP classes available to people in all counties, as well as classes that serve particular groups (eg, employer-based classes) that are not included in this analysis. Thus, availability of classes reported here is an imperfect proxy measure of accessibility. Also, we used county-level incidence of diagnosed diabetes overall as a proxy for determining where programs are needed, as that is the data available, although about 90% to 95% of diagnosed diabetes cases are type 2.

## Action

The maps highlight areas of need for LCP classes at the county level in the context of diabetes incidence rates and socioeconomic status. Only 17% of counties with the highest diabetes incidence and 10% of counties with the most socioeconomic disadvantage had a publicly available class location. Policy makers, program planners, and organizations engaged in expanding the availability of the National DPP LCP, including cooperative agreement grantees, can use this information to prioritize locations for additional programs, especially for identifying priority populations who are under-represented relative to their estimated numbers and disease burden.
